# The Reconstruction of Wolverhampton Hospital

**Published:** 1913-05-31

**Authors:** 


### THE RECONSTRUCTION OF WOLVERHAMPTON HOSPITAL.

### Mr. J. S. Neil, Secretary, on the Transition.

THE problem of adapting the original buildings of the Wolverhampton and Staffordshire General Hospital to the modern standard has long been pressing on the attention of the board of management. The present main building, built in the Italian style, dates from 1849, and as the problem of its reconstruction is one of exceptional difficulty, now that the new plans have been not merely decided upon but are actually being carried out by degrees, our Commissioner visited Wolverhampton, and through the courtesy of Mr. J. Stephen Neil, the secretary and house governor, made a tour of the building and heard how the difficulties of reconstruction are being overcome.

"The first and most general difficulty," Mr. Neil began, "is one that is inherent in any scheme of gradual adaptation—namely, to secure uniformity of standard throughout the additions. We have in the past often had to content ourselves with makeshifts, so far at least as the main original building is concerned, but it is extremely difficult to modify an old structure without falling foul of other up-to-date principles of construction in the process."

"When did the actual reconstruction begin?"

"Its necessity had long been recognised, but it was not till February 1910 that, after two years' work, the reconstruction committee submitted their final report and plans. The complete scheme, which is estimated to cost £54,100, comprised as its main features two ward blocks at either end of the main building providing an aggregate of seventy-two beds; one block of two wards with twenty-two beds apiece; a casualty department, with a children's ward over it; an out-patient department; a laundry and boiler-house; a new isolation block to take the place of our present old and unsuitable one; and certain important repairs and alterations to the old buildings. At present one of the two ward blocks which head this list has been built, and was opened last November, being known as the King Edward VII. Memorial Wing, which is the local memorial to King Edward."

"The internal alterations to the main block are extensive?"

"So extensive that it was only after prolonged discussion that it was eventually decided not to rebuild the entire hospital. The old buildings suffer from the cardinal defect that the wards, which are some sixty years old, run parallel to the front of the building instead of at right angles to it. The result is that the wards have only a corridor between them into which they both ventilate. The only thing to do therefore was, as far as possible, to build new wards, and to convert the main building into an administrative block. At present, as you will have noticed, the secretarial offices are away from the main building, and have been made one with the Bell Medical and Surgical Reference Library. The latter was presented under a deed to the effect that proper accommodation should be found for it, and this will be given in the new administrative quarters. The library will be designed so as to be used for a lecture hall also. At the present moment we are engaged in altering the main building, and especially in cutting up its centre so as to form an up-to-date theatre unit by the addition of sterilising, dark, anæsthetic, and staff rooms.

Mr. Neil now led the way through the main building, where the sound of hammering was distinctly audible.

"You will notice," he explained, "that the original wards were a series of small rooms, and we have done what we could to secure satisfactory administration by knocking them into one as far as possible. This has inevitably had to be rather a patchwork process, as it has been impossible to get an uninterrupted view of the rooms so joined: stanchions and walls through which in some instances only a doorway could be made still record their original separation. Having seen the earliest wards, and the difficulties with them, a later ward block which it has been decided to retain has also its interest. In these wards, which are forty years old, the chief fault is obvious: the chimneys from the centre stoves go up in the form of pillars to the ceiling, thus obstructing the view and occupying valuable air-space. But, as you see, there is a window between each bed, and sanitary spurs with cross ventilation between them and the wards. Now we come to the first section of the reconstruction scheme—that is to say, to the Edward Ward—and a description of it may serve as typical of the general improvements aimed at. Each new ward will, like this, accommodate eighteen beds, and be twenty-six feet wide, with a teak floor and teak doors made with a single flat surface. The walls are of white enamel with a tiled dado, which, incidentally, prevents any chance of the beds damaging the wall surface. Here again is a radiator system of heating supplemented by two central stoves with a down draught, the flues, of course, running beneath the floor."

"What novelties in details have you here?"

"Well, the lockers may be described as home-made, for they are my design, and consist, as you see, of teak with a glass top, a surrounding rail, and a drawer with a cupboard beneath. The cost worked out at £3 5s. each. In the sanitary spurs you notice the terrazzo floors, and this new device, a bed-pan flusher. Instead of washing the bed-pans in the sinks, this flusher which is placed on the wall as if it were a clock, has a glass face, which opens to receive the bed-pan on its end against the wall. When the door is shut water is admitted from a flushing cistern and flushes the pan in a moment, the water draining off with its own trap in the usual way. It not only is much safer for the nurses, but it removes the chance of breakage which must be run wherever heavy vessels are washed up in sinks. The flusher is a Jennings patent, and costs about £28. In the bathroom in the opposite spur you will notice that the bath is not fixed, but has a wheel at the opposite end to that of the taps, and so swings on a pivot. This is one of Dr. D. J. Mackintosh's ideas, which I saw at the Western Infirmary, Glasgow. When the bath is not being used it can be placed against the wall. When you want to use it it can be moved so that one can approach it from either side. In between the two sanitary towers is a deep embayed balcony, on to which beds can be wheeled. The teak seats on the balconies have an extension which may be placed alongside to increase the breadth of the seat, so that a mattress can be placed on it and the patient lie at full length. This also is a "home-made" contrivance. By the way, I should have drawn your attention in the bathroom to the open gulleys underneath the two washhand basins, which do away with some of the piping in the room itself. In the bathrooms attached to the old wards we still have some wonderful examples of good and solid work in the Rufford earthenware baths, which have been in use for forty years and show no sign of wear. The firm is still in existence. Later models may have the virtues of convenience and mobility. They will hardly wear better than these."

"In addition to the reconstructed theatre, what else is being done at the moment?"

"Balconies and fire-escape stairways are being built into the men's surgical and accident ward. In addition, the old coachhouse and stable has been converted into a motor ambulance house. At present we have an old horse ambulance, which is hardly sufficient to cope with the work required of it, for the patients come to us from a very wide area, extending as far as Stafford in the north, Wellington in the west, and Aldridge in the east, a circle, in fact, thirty-two miles in diameter. To give you an idea of the amount of work which the horse ambulance has got through, I may add that it was sent out 256 times last year. Thanks to the generosity of the local police force a motor ambulance is now due for delivery. This has become an urgent need, but the satisfaction which is keenly felt on this head makes us realise that probably the advertisement of the hospital which the motor ambulance will be may lead to its being sent for in unnecessary cases. Whether this proves so, and how to avoid its occurrence, only experience can decide."

"Want of money prevents the completion of the reconstruction?"

"Yes; but there are difficulties of site. The ground occupied by the hospital is triangular in shape, the apex being at an extremity of the front of the institution. This corner, however, has been leased, and the lessee is insisting on his rights to remain to the end of his term, though, according to the suggested plans, the corner now occupied by the lessee is destined for the new boiler-house. We are in the awkward position of having had to add on a boiler for the King Edward Wing to the already very small and crowded boiler-house quarters, and we cannot build any new wards until a boiler-house is built. Our steam pressure is insufficient to supply an extra block, and has had already to be supplemented. The scattered units that at present exist are, of course, a wasteful and unsatisfactory system; so that the provision of a new boiler-house is perhaps the most important of impending features to be undertaken. As the corner destined for it will not be free for building till the end of the tenancy—that is to say, till 1917—the question has to be considered whether enough space could not be found to build the boiler-house elsewhere in the meantime, and eventually, perhaps, to transfer the isolation block to this corner. As to the financial aspect of the question, the position is this: What we really require is a man of wealth and generosity enough to set a splendid example by giving us, say, £10,000; that would raise a large amount of enthusiasm, and his example would be followed by many subscribers of smaller sums. The difficulty is to find such a man. Because, odd though it sounds at first, while there are many men in the county of wealth and generosity, there are very few who could put their hand on so large a sum. The wealth of the Midlands is for the most part seen in capital expenditure on plant and factories. So that a man may be estimated at his death as worth half a million, and yet never have enjoyed during his lifetime an income of more than a few thousand pounds. The moment the factory stops, or the machines cease working, his perhaps immense capital may be reduced to the value of "scrap-iron." It is not money, but unrealised capital that many wealthy people here possess. That is why it is so hard to find a benefactor on the scale that we need."

"You are building a new laboratory?"

"Yes; that is a scheme for the immediate future. The plans for this, which, like those for all the reconstruction, have been drawn up by Mr. Arthur Worrall, a Wolverhampton architect, provide for a bacteriological and pathological laboratory in a separate unit to the north-west of the hospital. The laboratory is intended not merely to do all the work connected with the hospital, but also to undertake examinations and investigations for neighbouring practitioners as well. What happens at present is that whenever a practitioner requires an examination to be made his specimens are sent either to London or to Birmingham. A fully-equipped laboratory in connection with this hospital will, therefore, be of the utmost use to the hospital and the locality, and will enhance the reputation of the institution. It is believed also that the laboratory will be able to pay its way. But in order that these objects may be attained the first essential is to secure an absolutely first-rate man for the post. Before deciding on the plans we consulted Professor James Beattie, and after considerable care in selection have appointed Mr. William Boyd, the bacteriologist at Lancashire County Mental Hospital, as bacteriologist. He is to take up his duties on June 1, and we hope that the new laboratory will be ready in a few months' time. The necessary fittings have all been purchased. The unit will consist of a laboratory, a pathologist's private room, where the more delicate instruments can be kept, and a separate room for the autoclave sterilisers and incubators. This is the chief new departure in the reconstruction scheme, and one which not merely is designed to bring the institution up to date but to extend its activities in a new direction."

"There is also to be a new out-patient department?"

"Yes; notwithstanding the Insurance Act, we have found no reason to modify the department. As a matter of fact, from a census of patients that covered a period of three weeks I found that only 25 per cent. of our patients are insured. When you take away women, those under sixteen, those over age, and persons who are working for themselves, of whom we have a sprinkling, the proportion covered by the Insurance Act is not necessarily large, though it varies considerably in different districts."

"Have you an almoner here?"

"No. The fact is that the large majority of our patients come to us through various works with a Hospital Committee well acquainted with the position that each employee holds, and as these works possess tickets, for we have the ticket system here, it would be useless for a man in a superior position to apply for one, since it would be at once refused him. The ticket system, in fact, has proved of value to the hospital in the case of misunderstandings, for the hospital authorities have been able to point out that the giving of tickets is conditional on subscriptions and certain understandings, and that where these are not forthcoming or fulfilled, tickets may be unobtainable, and persons in consequence be prevented from becoming patients. No urgent case is turned away through want of a ticket."

"Are improvements also intended for the domestic staff?"

"Yes; it is impossible at present to accommodate them elsewhere than in the basement of the main building, which you have seen. But the allocation of the rooms at their disposal is being changed, so that they can have a more comfortable and better lighted dining room than they have at the moment."

